# Book Reviews

**Published:** 1899-03

**Authors:** 


### *BOOK REVIEWS, PAMPHLETS, EXCHANGES.*

---

### *BOOK REVIEWS.*

[Contributions solicited for review.]

---

Ocular Therapeutics for Physicians and Students. By J. W. Max Ohlemann, M.D., of Minden, Germany. Translated and edited by Chas. A. Oliver, A.M., M.D., of Philadelphia. Published by P. Blakiston's Son & Co., Philadelphia. Price $1.75.

This little book has just been issued from the above well-known publishing house. It is neatly gotten up in an attractive binding, and the paper upon which it is printed is excellent in quality. As for the work itself, we must confess that we do not exactly see what place it is expected to occupy, or even to fill. It certainly contains a great deal of matter that is valuable, but the same thing can be found in almost any good text-book on the eye. It appears to us, and we believe that is the intent of the book, that the author has simply compiled together those prescriptions which are most frequently used in certain acute or chronic inflammatory diseases of the eye by the most prominent ophthalmologists on continental Europe, especially in Germany. In other words, it is a small-sized manual on diseases of the eye, arranged differently from the usual methods. The author has done his work well, and has treated the various subjects in a very practical and clear manner. In treating different diseases of the eye one is glad to know sometimes how the treatment is conducted by various prominent ophthalmologists, and in this little book can be found the peculiar ideas of the best German men. The translator, Dr. Riesman, has evidently done his work well.

It is certainly a handy book for all ophthalmologists to possess, as it epitomizes ocular therapeutics in a clear manner.

A TEXT-BOOK OF OBSTETRICS. By Boston Cooke Hirst, M.D., Professor of Obstetrics in University of Pennsylvania. With 653 illustrations. Published by W. B. Saunders, Philadelphia.

Professor Hirst has succeeded in giving to the medical profession one of the best works on obstetrics that it has been our pleasure to see. We predict that it will have a large sale, and certainly it will be well deserved. The work is dedicated to Dr. Richard A. F. Penrose, and the author has a very modest preface as to his reasons for producing a new work on obstetrics. There is a great deal in this book to commend itself, both to the student and practitioner of medicine. In the first place, we like the arrangement; in the second place we like the clearness with which the various subjects are discussed, and in the third place, because of the beautiful illustrations, which are so needful in a work of this kind. The author's illustrations are, in many cases, original, taken from his own practice, and the colored plates descriptive of pathological conditions taken from life have been executed by Miss Margaretta Washington, an artist of exceptional ability in this line of work. Wherever a subject is discussed which, by its nature, is difficult to understand, the author has produced an illustration which will, in every instance, aid the student at a glance. This is especially true concerning the excellent illustrations given in the application of forceps. The chapter dealing with the management of the child after birth is filled with practical matter. We congratulate the publishers also on the excellency of its production.

---

ANNUAL AND ANALYTICAL CYCLOPÆDIA OF PRACTICAL MEDICINE. By Charles E. deM. Sajous, M.D., and One Hundred Associate Editors. Published by The F. A. Davis Company of Philadelphia and New York.

This is the second volume of the Annual for 1899, the first having already been reviewed. In a great many ways we like the new method of arrangement better than we did the old style. In the new arrangement the various subjects treated are placed alphabetically, instead of in one department, as previously. This volume takes us from Bromide of Ethyl to and through Diphtheria. We can unhesitatingly say that this work produced under the direction of Dr. Sajous, is the most valuable work that a physician can yearly place in his library. It epitomizes the whole domain of medicine, not only having the various subjects treated by men well capable of handling the same, but the literature is given as an appendix of all that has occurred of importance during the previous year. The illustrations are superbly produced, and in many cases they are chromo-lithographs. Some of the articles need especial mention in this volume, among which might be included "Cerebral Hemorrhage," by Dr. Wm. Browning; "Diabetes," by Professor Lepine, of Lyons, France; "Deaf-mutism," by Dr. Holger Mygind, of Copenhagen. It would be difficult to single out articles better than others, so excellent are they all.

The publishers are to be congratulated upon the style of binding and the excellency of the general make-up.

---

ON THE ORIGIN AND PROGRESS OF RENAL SURGERY. With Special Reference to Stone in the Kidney and Ureter. By Henry Morris, M.A., M.B., London, F. R. C. S., Senior Surgeon to the Middlesex Hospital; Examiner in Surgery in the University of London, etc. Published by P. Blakiston's Son & Co., Philadelphia. Price, $2.00.

This is one of the most valuable little works that has been issued from the press for some time. The book represent the Hunterian Lectures delivered by Dr. Morris for the year 1898. There are 285 pages, divided into four lectures, the last lecture not having originally been a part of the Hunterian Lectures, but which was later published in the *Edinburgh Medical Journal.* The scope of its contents may be readily seen from the headings of the different lectures as follows:

Lecture 1. On the Origin and Progress of Renal Surgery and the Conservative Tendency of its Recent Development.

Lecture 2. Renal Calculus.

Lecture 3. Fistula caused by Renal Calculus.

Injuries of the Ureter.

In addition to a thorough discussion of the subject as shown by these Lectures, there is appended a table of 267 operations on the kidney, which the author himself has performed. The illustrations are excellent throughout, and we consider it an unusually clear exposition of the present status of kidney surgery. To one who pursues to any extent this line of work this present little volume will prove of great value.

---

SELF-EXAMINATIONS FOR MEDICAL STUDENTS. Published by P. Blakiston's Son & Co., Philadephia. Price 10 cents.

This is a very neat little book of 189 pages, small enough to be carried in the vest pocket. It contains nothing but questions on the various subjects of medicine upon which a student must stand in his examination for a diploma in medicine. Opposite each page there is a blank sheet where annotations or any other memoranda may be placed. The book, of course, is intended for medical students. As such, it certainly has distinct value, for quizzing is the method *par excellence* adopted in preparing for any examination, which means also the proper understanding of the subject. Self-quizzing by means of a book like this is obliged to be of great help to students.

---

REPORT OF THE COMMISSIONER OF EDUCATION. For the Year 1896–7. Volume 3, containing Parts 2 and 3.

This volume, like the one previous, deals mostly with the problems of education. It considers the common school system in all the States in the Union. It also discusses normal schools and higher education. This work is especially valuable to those whose work lies in the line of pedagogy.

---

TRANSACTIONS OF THE MEDICAL SOCIETY OF THE STATE OF NORTH CAROLINA FOR THE YEAR 1898.

These Transactions show an excellency which is charateristic of the State of North Carolina. They are well gotten up and contain a number of important papers. We are always glad to receive these Transactions.

---

WE have received an "Adult Diet List," compiled by C. S. Millet, M.D., of Brockton, Mass. It is in the shape of a pad, so arranged that you can easily tear off a sheet and give it to the patient for reference. One side of the sheet contains a parallel list designated as "Eat" and "Try Cautiously," containing numerous articles of diet placed under these two heads. On the reverse side of the same sheet is a long list of eatables which one must "avoid." The author is treading in deep water, and we are afraid that he has undertaken a difficult task which not only one man, but many men cannot solve. Clinical experience has demonstrated the fact that every man is a "law unto himself," and that you cannot lay down fast rules for the guidance of all. All articles of diet do not have the same digestibility with all people, hence this list would be only valuable in bringing before the eye a quick, ready reference to the various articles of diet.

---

### MAGAZINES AND ANNOUNCEMENTS.

---

Messrs. E. B. Treat & Co. announce the early publication of *The International Medical Annual*, 1899. The publishers intend to make this issue more complete than ever. There will be a number of special articles by prominent physicians. It will be a splendid work for reference as to what was accomplished in medicine during the year 1898.

---

*Southern Home Journal*, published in Jackson, Miss., is an exceedingly attractive and entertaining little journal, and its editor, Miss May, is to be congratulated upon the success of her labors.

---

The February *Ladies' Home Journal* offers more than the expected variety of literature and pictorial features. It opens with an article by Mrs. Ballington Booth, taking the reader through State prisons, pointing out the awfulness of prison life, and the hopelessness of a released prisoner's efforts to gain unaided a place where he can get a livelihood. The story touches the heart, and will attract wide-spread interest. Mrs. Lew Wallace writes of "The Murder of the Modern Innocents," a powerful and convincing protest against the over-education of children. "The

Story of New York's Social Life" gives interesting glimpses of Gotham society, and "The Largest Ranch in the World" describes a Texas pasturage as large as two States of our Union. The three serials, "The Girls of Camp Arcady," Miss Wilkins's "The Jamesons in the Country," and "The Minister of Carthage," continue with dash and a successful interest.

